# Six-Year Complete Remission of Type-1 Diabetes Mellitus in an Adult Treated With Sitagliptin

**DOI:** 10.7759/cureus.34376

**Published:** 2023-01-30

**Authors:** Vânia Benido Silva, Maria Teresa Pereira

**Affiliations:** 1 Department of Endocrinology, Centro Hospitalar Universitário do Porto, Porto, PRT

**Keywords:** dipeptidyl peptidase 4 (dpp-4), honeymoon phase, low-carb, remission, type 1 diabetes

## Abstract

A “honeymoon” phase is a transient period of type 1 diabetes (T1D) remission, characterized by a significant reduction in insulin requirements and good glycemic control due to a temporary restoration of pancreatic β-cell function. This phenomenon occurs in about 60% of adults with this disease, is usually partial, and lasts for up to 1 year. We present a case of a 6-year complete remission of T1D in a 33-year-old man, the longest remission ever described in the literature to our knowledge. He was referred for presenting a 6-month history of polydipsia, polyuria, and weight loss of 5 kg. Laboratory studies confirmed the diagnosis of T1D (fasting blood glucose of 270 mg/dL; HbA1c of 10.6%, and positive antiglutamic acid decarboxylase), and the patient started intensive insulin therapy. After 3 months, a complete remission of the disease was assumed, he suspended insulin administration and since then, he has been under treatment with sitagliptin 100 mg daily, a low-carbohydrate diet, and regular aerobic physical activity. This work aims to highlight the potential role of these factors in delaying disease progression and preserving pancreatic β-cells when introduced at the time of presentation. More robust, prospective, and randomized studies will be needed to confirm its protective effect on the natural course of the disease and support its indication in adults with newly diagnosed T1D.

## Introduction

Type 1 diabetes (T1D) is a chronic disease characterized by immune-mediated destruction of functional pancreatic mass and subsequent insulin deficiency. The β-cell deterioration rate varies significantly between patients, leading to very heterogeneous forms of presentation and progression of this disease [1,2].

After diagnosis, about 60% of adults with T1D may experience a “honeymoon” phase, a transient period of remission characterized by a significant reduction in insulin requirements and good glycemic control due to a temporary restoration of pancreatic β-cell function [3]. Some factors have been indicated as potential predictors of the occurrence of this phase, namely disease presentation without diabetic ketoacidosis, older age, short duration of symptoms, and intensive physical exercise [4,5]. This remission is usually partial and lasts for up to 1 year [2]. The occurrence of complete remission is rare, with five more cases reported to date of total discontinuation of insulin for a period of more than 1 year [2,6-8].

Herein, we report a case of a 33-year-old man with T1D in complete remission for 6 years, keeping only under treatment with sitagliptin 100 mg daily, a low-carbohydrate diet, and regular aerobic physical activity.

## Case presentation

A 33-year-old Caucasian man was referred in December of 2016 to the endocrinology appointment for presenting a 6-month history of polydipsia, polyuria, and weight loss of 5 kg (previous body weight was 67 kg and BMI 23 kg/m^2^). He was previously healthy, and no stress events or infections were recognized prior to the onset of these symptoms. He did not practice any physical exercise. He denied tobacco or drug consumption and drank 20 g of alcohol per day. His family history was positive for diabetes mellitus (cousin with T1D mellitus diagnosed at age 33), and for autoimmune diseases (mother with hyperthyroidism due to Graves’ Disease and a paternal aunt with systemic lupus erythematosus).

Initial analytical investigation revealed a fasting blood glucose of 270 mg/dL, glycated hemoglobin (HbA1c) of 10.6%, C-Peptide of 0.29 ng/mL (Reference range (RR(: 0.3-2.3 ng/mL), and positive antiglutamic acid decarboxylase (anti-GAD65) of 11.81 U/mL (RR:<0.90 U/mL). Anti-insulin and anti-islet cell autoantigen (anti-ICA) antibodies were both negative. No elevation of ketonemia was documented. Morning plasma cortisol level and thyroid function tests were both normal. Thyroid cell autoimmunity was negative and celiac disease was excluded.

The diagnosis of T1D was performed and intensive insulin therapy with a basal-bolus regime (0.5 UI/kg/day per 60 kg) was started. Simultaneously, the patient adhered to a low-carbohydrate diet (estimated daily carbs ingestion of 80-100 g) and regular aerobic physical exercise (90 minutes of walking per day). Since then, a significant improvement in glycemic control and a progressive reduction in insulin requirements were verified. After 3 months of treatment, with HbA1c 5.4%, fasting blood glucose of 98 mg/dL and C-peptide of 0.5 ng/mL (RR: 1.10-4.40 ng/mL), a “honeymoon” period with complete remission of the disease was assumed. At this moment, the patient suspended insulin administration and started sitagliptin 100 mg per day, which he maintains until this day, combined with a pattern of a low-carbohydrate diet and daily physical activity.

Daily monitoring of interstitial glucose through a real-time flash glucose monitoring system and regular assessment of ketonemia levels were ensured. In 2021, 5 years after the diagnosis, the pancreatic autoimmunity study was repeated and the positivity of all autoantibodies was confirmed: anti-GAD65 102.3 U/mL (RR:<5.0), anti-protein tyrosine phosphatase (anti-IA2) 839.9 U/mL (RR <7.5), anti-ICA 11.9 (RR: <0.7), and anti-ZnT8 54.7 U/mL (RR: 0-14.9).

**Figure 1:**
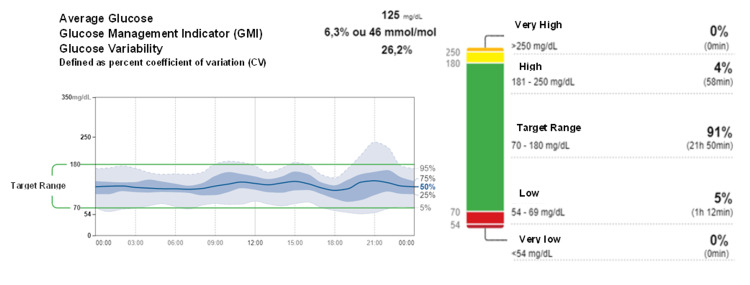
Ambulatory glucose profile of continuous glucose monitoring system from the last 28 days before the last visit

During these past 6 years, his HbA1c ranged between 4.8% and 5.7%, C-peptide between 0.29 and 0.94 ng/mL, and his BMI between 18.1 and 23 kg/m^2^. In the last follow-up, in November of 2022, the ambulatory glucose profile of the continuous glucose monitoring system from the last 28 days was assessed and the patient presented 88% of the time in a range (glucose between 70 and 180 mg/dL) and 3% of the time above range (glucose > 180 mg/dL), a glucose management indicator (GMI) of 6.1%, mean glucose of 117 mg/dL, and a coefficient of variation of 28.6% (Figure 1). The C-peptide was 0.65 ng/mL (RR: 1.10-4.40 ng/mL), fasting ketonemia ranged between 0.5 and 0.7 mmol/L (with the corresponding fasting glucose level between 102 and 155 mg/dL), and occasional ketonemia of 0.3 mmol/L (with a glucose level of 115 mg/dL).

## Discussion

We present a case of a 6-year complete remission of T1D in a 33-year-old man, the longest remission ever described in the literature to our knowledge. Since the suspension of insulin after 3 months of diagnosis, the patient has been under treatment with sitagliptin 100 mg daily, a low-carbohydrate diet, and regular aerobic physical activity.

There are five more cases reported with a “honeymoon” phase lasting more than 1 year [2,6-8]. As our patient, these cases occurred in individuals diagnosed in adulthood and without diabetic ketoacidosis (DKA), who were on a ketogenic diet and engaged in regular physical activity [2,6-8]. In addition, three of the five cases were also treated with sitagliptin [6,8].

Dipeptidyl peptidase-4 (DDP4) is a type II transmembrane glycoprotein involved in the metabolism of incretin hormones by degrading gastric inhibitory polypeptide (GIP) and glucagon-like peptide-1 (GLP-1) and is responsible for T-cell immune response due to T-cell activation, co-stimulation, proliferation, and migration [1,9].

Previous studies have reported that serum DDP-4 was upregulated in T1D, contributing to modulate inflammation and immune-mediated β-cell destruction [1,10]. Also, the peptide hormones GLP-1 and GIP seem to exert regenerative, anti-apoptotic, and proliferative effects in beta-cells mass [1]. Therefore, dipeptidyl peptidase 4 (DPP-4) inhibitors could have a potential role in delaying the progression of T1D to exert immunomodulatory effects [1,9,10]. Many studies have tried to confirm the impact of DDP-4 inhibitors in the preservation of beta-cell and, despite some incongruency, major results conclude in favor of its protective role [9,10].

Age at diagnosis and disease presentation seem to influence the time in remission [2]. Individuals who develop T1D in adulthood and without DKA have a greater pancreatic β-cells mass as well as a lower and more progressive rate of β-cell destruction [2]. The male gender also can be favorable to the occurrence of more prolonged remissions [3].

Another factor that seemed to predict the occurrence of this “honeymoon” period was the practice of physical exercise since T1D diagnosis [3,11]. Several studies have defended the role of regular aerobic physical activity in the preservation of β-cell mass through two mechanisms: by increasing cell proliferation and promoting blood elevation of growth hormone (GH), insulin-like growth factor-1 (IGF-1), and GLP-1; by reducing cell death by decreasing the levels of inflammatory cytokines (leptin and TNFa), increasing anti-inflammatory cytokines (adiponectin), modulating innate immunity, and reducing the destructive response to the β-cell [12]. Additionally, exercise promotes an increase in insulin sensitivity and a reduction in glucose and HbA1c levels [11].

Finally, the implementation of a low-carbohydrate diet (defined by the American Diabetes Association (ADA) as an intake of <130 g of carbs per day) has been shown to be associated with the immune reconstitution that occurs in the remission period [13]. This dietary pattern reduces glycemic variability and promotes a reduction in insulin requirements and an increase in peripheral sensitivity [10]. The main concern in adhering to this diet is the increased risk of developing DKA due to the potential elevation of serum ketone bodies [10]. However, with this case, we conclude that low-carbohydrates intake can be safe and without increasing ketonemia values, even in a condition of absolute absence of exogenous insulin administration and regular physical activity.

## Conclusions

To conclude, we report a clinical case of T1D in a man in complete remission for 6 years who has been maintained since diagnosis with sitagliptin, regular exercise, and a low-carbohydrate diet. Thus, this work aims to highlight the potential role of these factors in delaying disease progression and preserving pancreatic β-cells when introduced at the time of presentation. More robust, prospective, and randomized studies will be needed to confirm its protective effect on the natural course of the disease and support its indication in adults with newly diagnosed T1D.
